# Dielectric Measurement Based Deducted Quantities to Track Repetitive, Short-Term Thermal Aging of Polyvinyl Chloride (PVC) Cable Insulation

**DOI:** 10.3390/polym12122809

**Published:** 2020-11-27

**Authors:** Gergely Márk Csányi, Semih Bal, Zoltán Ádám Tamus

**Affiliations:** Department of Electric Power Engineering, Budapest University of Technology and Economics, H-1111 Budapest, Hungary; csanyi.gergely@vet.bme.hu (G.M.C.); semihbal@hotmail.com (S.B.)

**Keywords:** PVC insulation, low-voltage cables, insulation degradation, short-term thermal degradation, dielectric measurements, hardness testing, loss factor, voltage response measurement

## Abstract

The effect of short-term (3- and 6-h-long) periodic thermal aging was investigated at three different temperatures on PVC cables and PVC films. Three different temperatures (110, 125, and 140 °C) were used for aging PVC cables and one (110 °C) for PVC films. PVC films were prepared for the investigation containing 0, 30, 40, and 50 weight percent of dioctyl phthalate plasticizer (DOP). The effect of short-term thermal aging was monitored by electrical (dielectric spectrum and voltage response measurement) and mechanical (Shore D hardness) methods. From the loss factor measurements, different deducted quantities were calculated and compared with Shore D hardness, which has been shown to be a parameter reflecting the effect of short-term thermal aging on PVC insulation. The measurements revealed that Shore D hardness is not the best property for monitoring aging. Instead, increasing dissipated power and the shifting behavior of tan δ–frequency curves proved to be the best phenomena for assessing the impact of thermal aging. Simple deducted quantities may provide a basis for following short-term thermal aging.

## 1. Introduction

Due to strict regulations, the market share of low-smoke halogen-free cables is rapidly increasing. However, polyvinyl chloride (PVC)-insulated low-voltage (LV) cables are still prominent for electrical distribution and the safe operation of nuclear power plants (NPPs) [1,2,3]. Condition monitoring and an understanding of the degradation processes due to short-term thermal stress for low-voltage cables have been of interest to the nuclear industry because of qualification requirements [4,5]. New challenges brought about by distributed generation and novel appliances connected to the LV distribution grid (e.g., solar panels and electric vehicles) increase the need to extend these techniques to LV distribution cables. Connecting appliances to the low-voltage grid due to reverse power flow may cause the aggregated load to surpass the cable’s rated capacity in a way that might be undetected by a protecting fuse. These short-term overloads lead to temperature elevation above rated operating limits, which may lead to damage that can decrease the lifetime of cables [3,6]. If the temperature is followed using smart meters and the correlation with aging is known, a better condition monitoring system can be created [7], increasing the reliability of the LV distribution cable networks.

Many studies have shown that thermal aging affects the mechanical and electrical properties of cables [8,9,10,11,12]. Moreover, chemical and physicochemical measurement methods have also been demonstrated to be valid approaches for tracing structural changes occurring in the insulating material [13,14,15]. It has also been shown that the effect of even a few-hour-long overload can be measured and may degrade cable condition and change the dielectric spectrum of the insulation [16,17,18,19].

Correlating the dielectric spectrum of an insulation material and aging time is a complicated task. Analysis may require evaluation of the strength of the relationship between two quantities. To meet the requirements for calculating the correlation, it is necessary to order only one number to the dielectric spectrum curve. For this study, the correlation between short-term thermal aging of PVC insulation and several deducted quantities from the dielectric spectrum was assessed. To eliminate the effect of the complex structure of the cable and the presence of colorant additives [20], nine thermal aging periods were utilized for PVC films having four different plasticizer contents at 110 °C. Results and deducted quantities from the dielectric spectrum (i.e., tan δ vs. frequency curves, voltage response (VR), and Shore D measurements) were compared to reveal which marker shows strong correlation with short-term thermal degradation of PVC insulation.

## 2. Materials and Methods

### 2.1. Samples

#### 2.1.1. PVC Cable Samples

**Figure 1:**
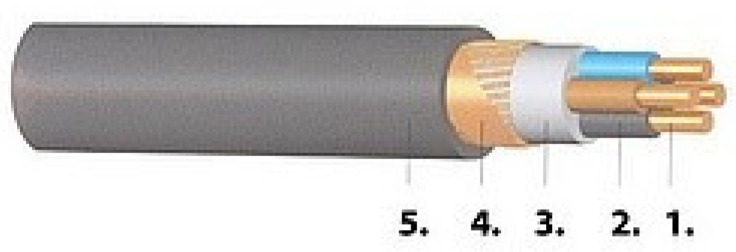
The construction of the cable: four copper conductors (**1**), PVC core insulations (**2**), filling material (**3**), copper wire and tape screens (**4**), and PVC jacket (**5**).

During the investigation, nine pieces of half-meter-long NYCWY 0.6/1 kV 4 × 10/10 mm^2^ low-voltage cables (Prysmian MKM Kft., Budapest, Hungary) were used. Figure 1 shows the structure of the cable.

**Figure 2:**
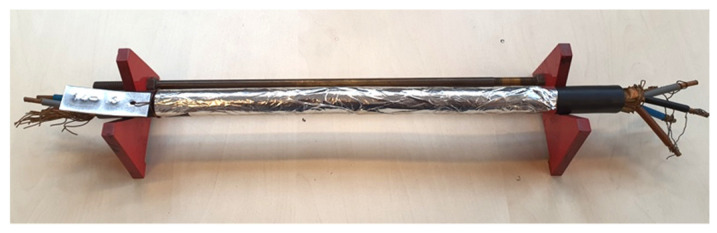
Cable sample prepared for measurement. The inner conductor is the screen tape connected to all core conductors; the outer one is an aluminum foil wrapped around the cable.

The core insulations were colored blue, black, grey, and brown. According to manufacturer information, the maximum operating temperature is 70 °C, while the conductors can heat up to 160 °C during short circuit. The manufacturer did not provide any information regarding thermal aging. To measure the cable jacket, inner and outer electrodes were prepared as shown in Figure 2. Inner electrode was created by connecting wire and tape screens to all core conductors. Outer electrode was created by wrapping an aluminum foil around each cable jacket.

#### 2.1.2. PVC Films

To eliminate the effect of the relatively complicated geometry, dying additives [20], and layered insulation structure and focus solely on the material itself, PVC films were prepared for the investigation. The samples had four different plasticizer contents: 0, 30, 40, 50 weight percent (wt %) and size of 100 mm × 100 mm × 1 mm. Each plasticizer group contained three samples. The elements used in the compound were the same as in [21], which are as follows: Ongrovil S-5070 type PVC powder (100 wt %, BorsodChem Zrt. PVC Manufacturing, Kazincbarcika, Hungary), lead stabilizer (3 wt %, Naftomix PBV 30 Akdeniz Chemson Additives A.G., Arnoldstein, Austria), stearic acid lubricant (0.3 wt %, Molar Chemicals Kft., Halásztelek, Hungary), and dioctyl phthalate plasticizer (DOP, KH Chemical BV, Scheepmakerij, The Nederlands) in the following weight percentages: 0, 30, 40, 50 wt %. Generally, a new PVC sheath of a cable has its Shore D hardness in the range of 35–45 [9,11]. The plasticizer contents were chosen in a way that one specimen group had almost the same Shore D hardness as a new cable sheath (40 wt %). Another specimen group had significantly lower Shore D hardness (30 wt %), and another one had significantly higher Shore D hardness (50 wt %). The compound did not contain dying additives since previous studies have revealed that these might have an effect on the dielectric properties of an insulation material [20]. DOPXX_Y_ denotes the *y*th sample containing XX wt % of the plasticizer (e.g., DOP40_1_ means the first sample of the group where 40 wt % of the plasticizer content was used in the PVC compound).

### 2.2. Thermal Aging

#### 2.2.1. PVC Cable Samples

The samples were organized into three groups denoted by 110 °C, 125 °C, and 140 °C. The names of the groups refer to the temperatures used during thermal aging. Each group contained three cable specimens. The steps of an accelerated aging cycle are as follows:

- Heating up the heating chamber to the required temperature and letting the temperature stabilize for at least an hour
- Putting the samples into the heating chamber for 3 or 6 h depending on the aging cycle
- Removing the samples from the heating chamber
- Placing the specimens in the laboratory where the temperature was controlled
- Letting the samples rest for at least 8 h

Hence, a complete accelerated aging cycle lasted for around 2 days.

The samples in groups 110 °C and 125 °C were exposed to eight cycles of accelerated thermal aging. The first six aging cycles were 3 h long. The last two lasted 6 h each. By the end of the investigation, both 110 °C and 125 °C groups reached 30 h of aging. It is important to note that after reaching 12 h in total aging (after aging period no. 4), the specimens were resting for around 2 months, although an aging round was performed in 2 days. Since the aging was performed in a ventilated chamber at high temperatures, the plasticizer loss is probably a diffusion-controlled process. Hence, the thermal aging may cause inhomogeneous plasticizer concentration in the PVC components of the cable (jacket, filling material, and core insulation), containing less plasticizer close to the surface. By introducing a 2-month-long resting period to the cables, inhomogeneous plasticizer concentration may have enough time to recover to homogeneous plasticizer concentration.

The cable specimens in group 140 °C were exposed to four periods of aging. The first two periods lasted 3 h; the second two lasted 6 h, reaching 18 h of total aging by the end of the investigation. In group 140 °C, the aging periods were not interrupted by any resting periods.

Equivalent aging times were calculated from the different aging temperatures to 110 °C, applying the Arrhenius equation. During the calculation, activation energy was set to 80 kJ/mol (0.829 eV) according to [22]. The equivalent aging times can be seen in Table 1.

#### 2.2.2. PVC Films

PVC films were organized into four different groups, namely, DOP0, DOP30, DOP40, and DOP50. All samples in a group contained the same amount of plasticizer. Each group was exposed to nine aging periods at 110 °C in a ventilated heating chamber (Binder GmbH, Tuttlingen, Germany). The first seven aging periods lasted 3 h, and the last two periods lasted 6 h, with 33 h of total aging. A 1-month-long resting period was applied for the DOP30, DOP40, and DOP50 groups. For the DOP30 and DOP40 groups, the resting period started after 15 h of total aging, while for the DOP50 samples, after 18 h. The purpose of including a resting period during aging was the same as mentioned in the case of cable samples. By letting the PVC films rest, the inhomogeneous plasticizer content could recover if diffusion-controlled plasticizer loss takes place during thermal aging.

All the samples were holed in the upper right and upper left corners. The holes were used to hook the samples to a metal rod that was fixed in the heating chamber. This way, the direct contact between metal parts and the samples could be minimized.

### 2.3. Measurement Methods

#### 2.3.1. PVC Cable Samples

A Bareiss HPE II Shore D durometer (Bareiss Prüfgerätebau GmbH, Oberdischingen, Germany) was used to measure mechanical parameters. The hardness was measured 10 times on each cable jacket after removing the aluminum foil and selecting the measurement spots randomly. These Shore D measurement results serve only comparison purposes because the thickness of the jacket reaches about 2 mm, meanwhile, for example, the ASTM D2240 standard requires at least 6 mm thickness of the inspected material.

**Figure 3:**
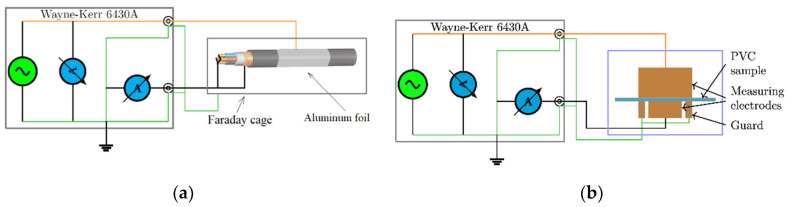
The measurement of tan δ by impedance analyzer: (**a**) measurement of cable samples in a Faraday cage; (**b**) measurement of PVC films by guarded electrode setup.

Tan δ vs. frequency curves were monitored by a Wayne–Kerr 6430A impedance analyzer (Wayne Kerr Electronics, West Sussex, UK). The connection of cable samples to the equipment can be seen in Figure 3a. The tan δ values were measured 14 times, increasing the frequencies logarithmically in the 20 Hz–500 kHz range at 5 V. During the measurement, the equipment was set to parallel R-C mode. The specimens were measured at 23 ± 1 °C.

**Figure 4:**
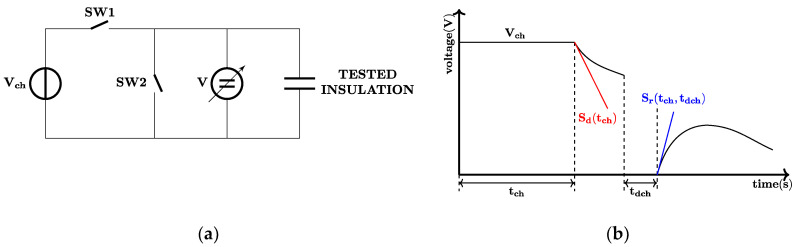
The voltage response measurement: (**a**) Schematic of the measurement. SW1 is used for charging, while SW2 is for discharging. During the measurement, both switches are in OFF position. (**b**) The timing diagram. Charging and discharging times are denoted by *t_ch_* and *t_dch_*, respectively. *S_d_*(*t_ch_*) and *S_r_*(*t_ch_, t_dch_*) are the slopes of decay and return voltages, respectively.

**Figure 5:**
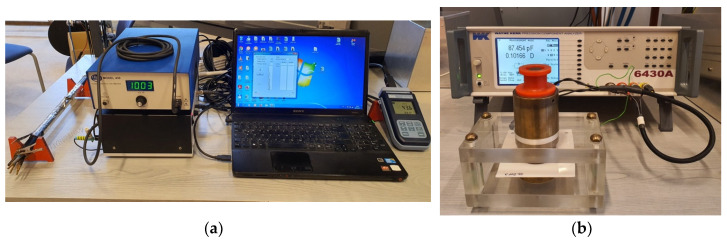
Measurement of dielectric properties: (**a**) voltage response measurement on a PVC cable sample; (**b**) measurement of PVC films by cylindrical copper electrodes by Wayne–Kerr 6430A impedance analyzer.

The equipment that were used for measuring voltage responses were developed at the Budapest University of Technology and Economics, Department of Electric Power Engineering. After a long duration (1000–10,000 s) of charging by DC voltage, VR method [23,24] measures the slopes of the decay voltage (*S_d_*) after disconnecting the insulation from the voltage source. Slopes of return voltages (*S_r_*) can be measured after a few seconds of short-circuiting the insulation. Figure 4 shows the schematic and timing diagram of the measurement method. For charging time, 2000 s was chosen, and charging voltage was set to 1000 V DC. The inner electrode (inner cores and wire and tape screens) was connected to 1000 V DC, and the outer electrode (aluminum foil) was grounded during the VR measurements (Figure 5a) to reduce electromagnetic noises. The specimens were measured at 23.35 ± 0.55 °C. Proper connections were tested before each electric measurement. During this investigation, only the cable jackets were measured.

#### 2.3.2. PVC Films

In the case of PVC films, the same measurements were performed as in cables with the same settings except for the following details.

The samples were measured by putting the films between two copper cylinders having 35.6 mm diameter each as in [21] (Figure 3b and Figure 5b). Due to the high temperature dependence of dielectric processes in PVC, the measurements were executed in an air-conditioned laboratory, where the temperature was 25 ± 0.5 °C.

Shore D measurements were executed in the following manner. In the case of plasticized specimens, the three samples of an aging group (e.g., DOP30) were stacked on each other and put onto the DOP0_1_ sample so as to reach a total thickness of around 4 mm. After carrying out 10 measurements on the topmost sample, the order of the plasticized samples was changed to have one unmeasured sample on top. In the case of nonplasticized (DOP0) specimens, only three samples were stacked. After measuring the hardness of the topmost sample, the order of the plasticized samples was changed to have one unmeasured sample on top. The reason for using only three samples instead of four is that the DOP0 specimens had ca. two times greater hardness than the plasticized samples. Hence, the contact between the table and the presser foot of the hardness tester was avoided.

### 2.4. Measured and Calculated Deducted Quantities for Following Aging

The tan δ as the function of frequency characterizes the dielectric loss of the material in a wide frequency range. Changes in material structure also change the shape or the position of tan δ(f) curves. Although these curves contain very detailed information about the dielectric loss of the material, the “shape changing” is not an exact parameter for monitoring degradation or for correlation analysis. Hence, deducted quantities were introduced and tested. The central loss factor (CLF) and central frequency (CF) are two deducted quantities that have been demonstrated to be valid approaches for condition monitoring [18]. Central loss factor, central frequency, and five more deducted quantities are defined below. In each case, the index *i* refers to the *i*th measurement carried out in the 20 Hz–500 kHz range.

#### 2.4.1. Central Loss Factor (*CLF*)

The *CLF* was calculated by summing up the multiplication of the logarithm of the frequencies by the measured tan *δ* values at given frequencies and dividing this sum by the sum of the logarithm of the frequencies, as can be seen in Equation (1).

(1) $$CLF=\frac{\sum_{i=1}^{n}log_{10}f_{i}\times tan\delta_{i}}{\sum_{i=1}^{n}log_{10}f_{i}}$$

#### 2.4.2. Central Frequency (*CF*)

The *CF* was calculated by summing up the multiplication of the logarithm of the frequencies by the measured tan *δ* values and dividing this sum by the sum of the measured tan *δ* values and using this as an exponent to base 10 as Equation (2) shows.

(2) $$CF=10^{\frac{\sum_{i=1}^{n}log_{10}f_{i}\times tan\delta_{i}}{\sum_{i=1}^{n}tan\delta_{i}}}$$

#### 2.4.3. Central Capacitance (*CC*)

The central capacitance (*CC*) was calculated by the same logic as the *CLF* values just substituting the tan *δ* values by the capacitance values as Equation (3) shows.

(3) $$CC=\frac{\sum_{i=1}^{n}log_{10}f_{i}\times C_{i}}{\sum_{i=1}^{n}log_{10}f_{i}}$$

#### 2.4.4. Capacitance × Log(Frequency) × tan *δ* (*CxlgFxLF*)

Another deducted quantity, the capacitance × log(frequency) × tan δ (*CxlgFxLF*), was calculated as shown in Equation (4) by multiplying the logarithm of the frequencies by the corresponding loss factor and capacitance values for each frequency, respectively, and summing them.

(4) $$CxlgFxLF=\overset{n}{\underset{i=1}{\sum }}log_{10}f_{i}\times tan\delta_{i}\times C_{i}$$

#### 2.4.5. Capacitance × Frequency × tan *δ* (*CxFxLF*)

The capacitance × frequency × tan *δ* (*CxFxLF*) was calculated as in Equation (4), but instead of using the logarithmic frequencies, the frequencies of the measurement were used.

(5) $$CxFxLF=\overset{n}{\underset{i=1}{\sum }}f_{i}\times tan\delta_{i}\times C_{i}$$

#### 2.4.6. Area of Capacitance Times tan *δ* at Logarithmic Frequencies (*Alog*)

A similar deducted quantity to the latter was calculated by basically integrating the tan *δ* times capacitance values at the logarithmic frequency scale. The integration was performed by calculating the area of rectangles and summing these areas up as Equation (6) shows,

(6) $$Alog=\overset{n-2}{\underset{i=1}{\sum }}\left(min\left(p_{i},p_{i+1}\right)+\left(max\left(p_{i},p_{i+1}\right)-min\left(p_{i},p_{i+1}\right)\right)/2\right)\times \left(log_{10}f_{i+1}-log_{10}f_{i}\right)$$

where *p_i_ = C_i_ × tan δ_i_* and *i* is the *i*th frequency in which the samples were measured but without the last one.

#### 2.4.7. Multiplication of Available Values: *CFxCLFxCC*, *CFxCLF*

By multiplying the *CF*, *CC*, and *CLF* values, another two deducted quantities (*CFxCLFxCC* and *CLFxCC*) were calculated.

#### 2.4.8. VR Measurements

From the VR measurement results, the initial slope of decay voltage (*S_d_*) and the initial slope of return voltage (*S_r_*) were used. *S_d_* is directly proportional to the specific conductivity of the insulating material, and *S_r_* is directly proportional to the polarization conductivity [25,26]. In this study, only the *S_d_* values were used in both cables and films since *S_r_* was demonstrated to have weak correlation with aging on cables [19].

## 3. Results—PVC Cable Samples

### 3.1. Shore D vs. Aging

**Figure 6:**
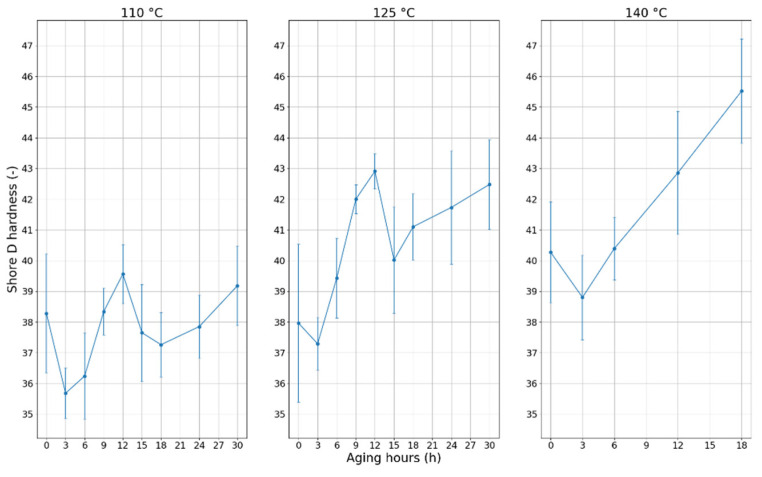
Changes of the hardness of PVC cable samples with aging time at each temperature.

The measurement results at each temperature can be seen in Figure 6. As in [11,18,19], the hardness of the jacket after an initial drop increases, and this behavior can be inspected at each aging temperature. The effect of the 2-month-long rest was clearly visible in the case of 110 °C and 125 °C: the values after 12 h showed nearly the same behavior as the results after 0 h. In both cases, after an initial drop, an increasing trend could be inspected.

### 3.2. Loss Factor (Tan Delta) vs. Aging

**Figure 7:**
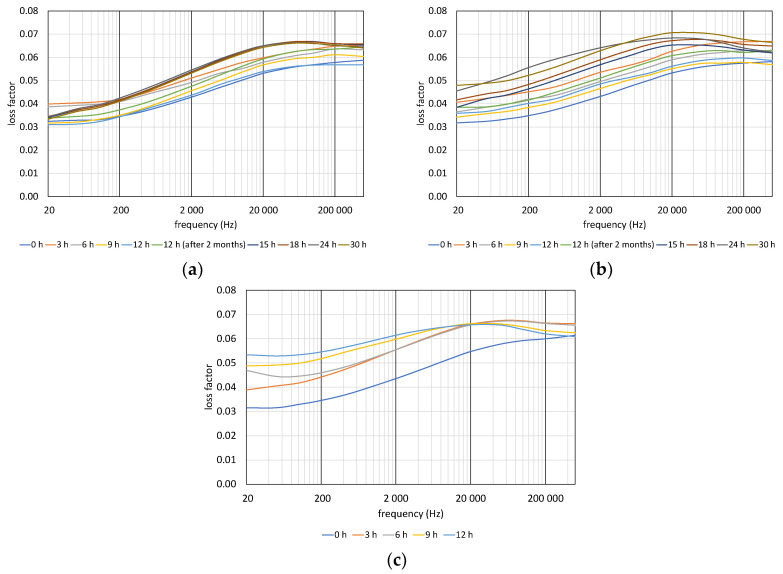
Changing of loss factor with aging time: (**a**) 110 °C, (**b**) 125 °C, (**c**) 140 °C.

The results of loss factor measurements can be seen in Figure 7. The effect of the 2-month resting of the samples can be clearly seen in Figure 7a,b. The tan delta values increased at each frequency. The general trend of loss factor curves can be seen in Figure 7c. The loss increased, and the curve shifted leftward, so the peak of the loss moved to lower frequencies.

### 3.3. Slope of Decay Voltage (S_d_) vs. Aging Time

**Figure 8:**
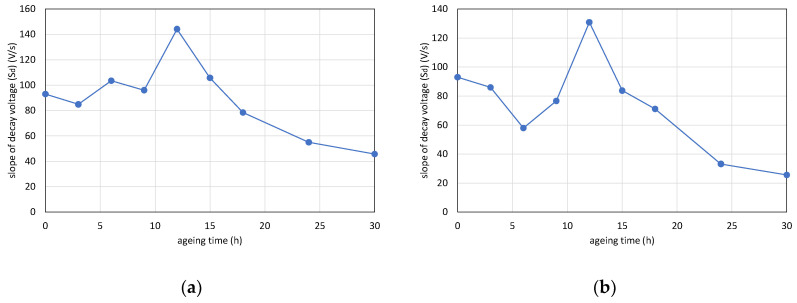

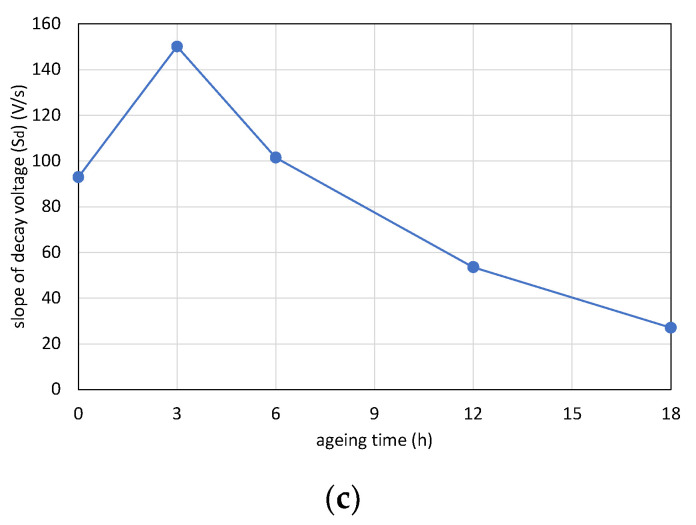
Changing of slope of decay voltages with aging time: (**a**) 110 °C, (**b**) 125 °C, and (**c**) 140 °C.

The results of the voltage response measurement with aging time can be seen in Figure 8. In the case of the 110 °C and 125 °C aging temperatures, similar to the Shore D hardness values (Figure 6), the *S_d_* values started to decrease in the first few aging periods. Then, the values increased until reaching a peak. Reaching the peak, the slopes of decay voltages decreased again. At a 140 °C aging temperature, the *S_d_* reached a peak after 3 h of aging. Then the results followed a decreasing trend.

### 3.4. Comparison of Deducted Quantities

The previous study of the authors claimed that central loss factor and central frequency values are relatively good deducted quantities for monitoring thermal aging for PVC-insulated cables. However, so far it has not been possible to decide whether these parameters are better than Shore D hardness or not [18]. Otherwise, as Figure 7 shows, the changing of loss factor curves is clearly visible, but the calculation of correlation with aging requires numerical values. Therefore, for correlation analysis, ordering only one numerical value for each tan delta vs. frequency curves is necessary.

The results of the correlation analysis of deducted quantities are in Table 2.

The correlation coefficients were calculated between the proposed deducted quantities and aging time. These rows are denoted by “Aging t.” in Table 2. Previous studies have shown that the Shore D value correlates well to the degree of thermal aging [9]. Hence, other deducted quantities were compared with the Shore D values (“ShoreD” rows in Table 2) and the changes in Shore D values (“Diff.”). Differences (“Diff.” in Table 2) show how well the deducted quantities follow the changes of Shore D by thermal aging. Differences were calculated as the differences of the Shore D values between two measurement points divided by the aging time between the two points. Correlation coefficients were calculated between the differences values of the Shore D results and other deducted quantities. The measured data were normalized by dividing each value by the measured data at 0 h of aging. This way, every deducted quantity starts from the value of 1. Table 2 shows the correlations between the deducted quantities by (equivalent) aging time, Shore D, and differences values in case of equivalent aging times at 110 °C and all aging temperatures as well.

#### 3.4.1. All Measurements Converted to Equivalent Aging at 110 °C

**Figure 9:**
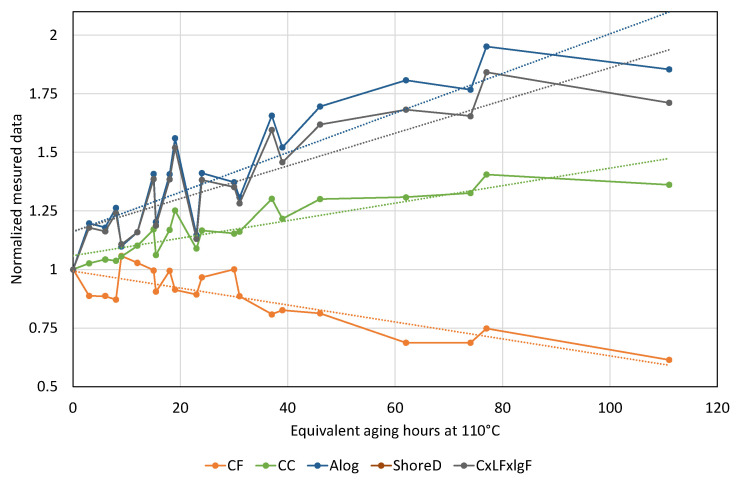
Deducted quantities with the highest correlation coefficients for following aging at 110 °C. The dashed lines are the corresponding linear regression lines of a given deducted quantity.

As Table 2 shows, Shore D values follow aging well since the correlation between the equivalent aging times is 0.853 considering 80 kJ/mol (0.829 eV) activation energy for aging. However, four deducted quantities proved to have better or comparable correlations with Shore D: Alog (0.875), CC (0.874), CF (−0.853), and CxLFxlgF (0.849). These deducted quantities are plotted in Figure 9. It can be seen that all deducted quantities had higher slopes by aging than Shore D hardness. These deducted quantities had higher sensitivity to thermal aging than Shore D hardness.

Examining the differences, the order is CFxCLF (−0.897), CxFxLF (−0.844), CFxCLFxCC (−0.835), and CLF (−0.831), but CxlgFxLF (−0.765) and Alog (−0.753) are also worth mentioning. If both the aging time and the cochanging with Shore D hardness are considered, the more reliable parameter for following aging and hardness together is Alog, and the next one is CxlgFxLF.

#### 3.4.2. Measurements at 110 °C

Because 110 °C is the lowest temperature for aging, the measured values characterize more the early period of aging. The results show that the best parameters for monitoring aging time are CC (0.877), CFxCLFxCC (0.869), and CxFxLF (0.849), but CFxCLF, Alog, and CxlgFxLF are also above 0.8 correlation. All of these quantities are significantly better than Shore D, which has a correlation of only 0.432 to the equivalent aging time. The differences column shows that CLF is the best choice by −0.941, but CxFxLF, Alog, and CxlgFxLF are also above −0.86 correlation. This result means that these values follow well the changing of Shore D hardness by thermal aging.

#### 3.4.3. Measurements at 125 °C

In the case of the 125 °C group, the best deducted quantities for following aging time are CC (0.984), Alog (0.959), CxlgFxLF (0.957), CxFxLF (0.935), and CF (−0.898). Nevertheless, CFxCLFxCC (0.835) and CLF (0.821) also provided better results than Shore D (0.721). The differences column shows interesting results since the highest correlation (Alog, −0.754) was lower than the differences in the case of 110 °C. Other quantities worth mentioning are CLF (−0.739) and CxlgFxLF (−0.707).

#### 3.4.4. Measurements at 140 °C

Since at 140 °C only four measurements were carried out, these correlation values have to be considered accordingly. This temperature was the highest among the three aging temperatures. Therefore, the measurement results of this temperature characterize more the effect of long-term thermal aging. Three parameters followed aging better than Shore D (0.932): CLF (−0.98), *S_d_* (−0.98), and CF (−0.962), while Alog was the first below the level of Shore D, having a 0.86 correlation coefficient to aging time. The differences in all but two cases were higher than 0.9 considering unsigned correlation coefficients.

## 4. Results—PVC Films

### 4.1. Shore D vs. Aging

**Figure 10:**
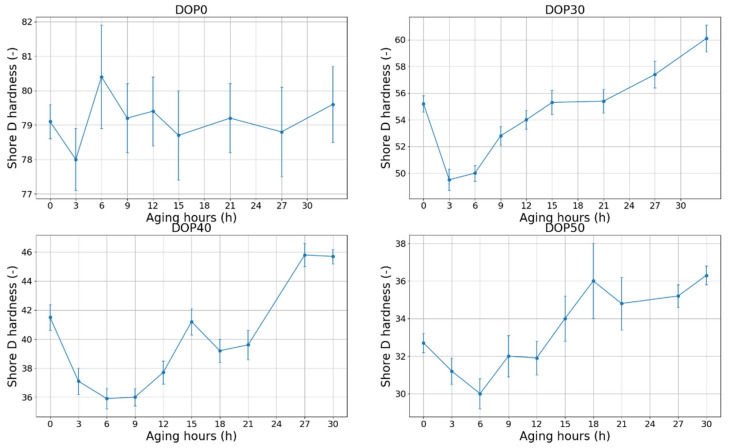
Changes of hardness of PVC cable samples with aging time at each temperature [27].

The results of the hardness measurements can be seen in Figure 10. All plasticizer contents showed the same behavior as in Figure 6: after an initial drop, the hardness increases and the effect of the 1-month-long rest is also clearly visible. However, in the case of the nonplasticized DOP0 specimens after the initial drop and increment, the hardness slightly decreased and remained constant. Another difference that is worth mentioning is that the effect of resting is becoming less significant by having less plasticizer content. In the case of DOP50, 15 h of aging was needed to reach the initial hardness before the resting (18 h of total aging). Meanwhile, the times were only ca. 9 h and 6 h in the cases of DOP40 and DOP30 samples, respectively.

### 4.2. Loss Factor (tan delta) vs. Aging

**Figure 11:**
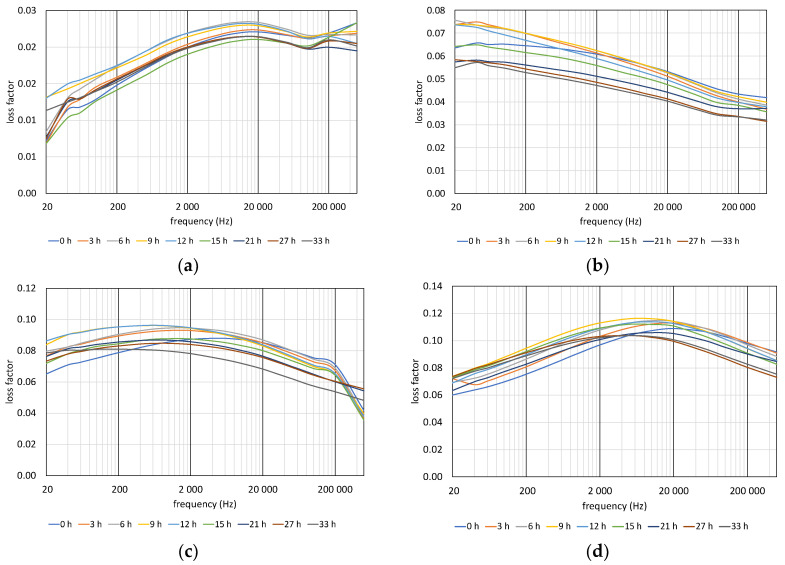
Changing of loss factor with aging time of PVC films: (**a**) DOP0, (**b**) DOP30, (**c**) DOP40, and (**d**) DOP50.

The results of loss factor measurements of PVC films are shown in Figure 11. As the figure shows, the shapes of loss factor vs. frequency curves and the magnitudes of the dielectric loss strongly depend on the amount of contained plasticizer. By increasing DOP content, the tan δ values increase. In the case of DOP30, the loss factor curves are decreasing monotonically by frequency, while the other curves are concaves. The peak of the dielectric loss is around 20,000 Hz in the case of non-plasticized (DOP0) samples. In the case of DOP40, the peak values are around 2000 Hz, and around 8000 Hz in the case of DOP50 samples.

Shifting of peaks with aging can also be observed in the case of DOP40 and DOP50 samples. In both samples, the peaks shifted leftwards by thermal aging. As a result of the first four aging periods, the magnitude of the peaks follows an increasing trend. As aging advanced, the magnitude of the peaks decreased.

### 4.3. Slope of Decay Voltage (S_d_) vs. Aging Time

**Figure 12:**
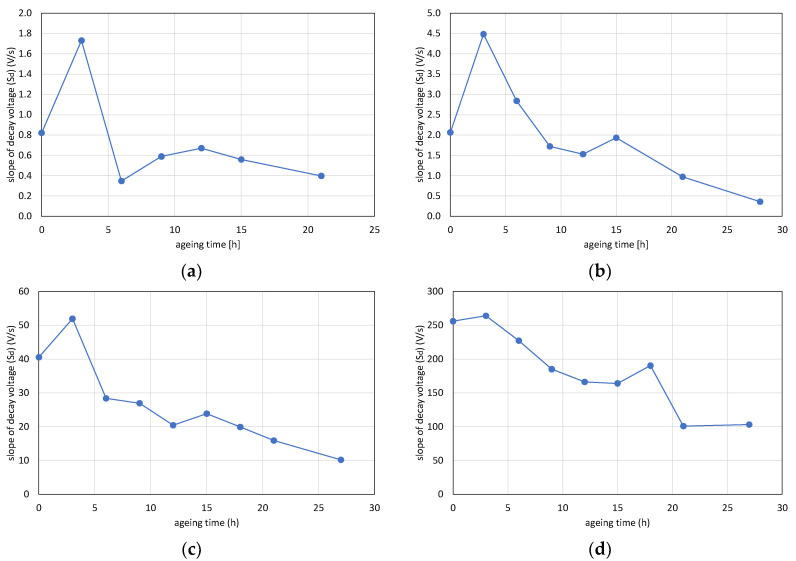
Changing of slopes of decay voltages with aging time of PVC films: (**a**) DOP0, (**b**) DOP30, (**c**) DOP40, and (**d**) DOP50.

The results of slopes of decay voltage measurement are shown in the diagrams in Figure 12. The slopes of decay voltages increased by the amount of plasticizer content. Hence, the higher the plasticizer content is, the higher the conductivity is. The trend of the curves is very similar to each other. After the first aging period, the *S_d_* values increased, then converted to a decreasing trend with aging. The effect of 1-month-long resting can also be observed: the conductivity increased after letting the samples rest (15 h DOP30, DOP40; 18 h DOP50).

### 4.4. Comparison of Deducted Quantities

The calculated correlations between the deducted quantities are shown in Table 3. The meaning of the rows is the same as in Table 2.

#### 4.4.1. Nonplasticized Specimens

The best quantities for following aging in the case of nonplasticized PVC (DOP0 samples) have been CxFxLF (−0.865), CC (−0.715), and CxLFxlgF (−0.686). It is important to note that Shore D hardness shows the lowest correlation of all the results. This is not surprising since hardness correlates mostly with the plasticizer content. It is important to note that thermal degradation also increases the hardness of PVC, although the total aging time is not long enough for significant dehydrochlorination.

When the correlation to the Shore D hardness is examined, the best quantity is *S_d_* (−0.776). All the others are below 0.5 correlation. Examining the differences, the correlations improved significantly compared with the correlation with hardness, lifting 6 quantities out of 11 above 0.8 correlation. However, in this case, it has to be neglected since the changes in hardness were also negligible.

#### 4.4.2. Plasticized Specimens

The Shore D hardness followed well the thermal aging in the case of plasticized specimens reaching 0.81, 0.724, and 0.836 correlations on DOP30, DOP40, and DOP50 samples. However, four better quantities have been found to be better at all plasticizer contents. In the order of (DOP30, DOP40, DOP50) CxFxLF (−0.935, −0.929, −0.958), CC (−0.843, −0.905, −0.95), CFxCLFxCC (−0.867, −0.869, −0.936), and CFxCLF (-0.811, -0.831, -0.829). Moreover, in the case of lower plasticizer contents (30 and 40 wt%), CLF (−0.95, −0.879, −0.69), CxLFxlgF (−0.935, −0.875, −0.771), and Alog (−0.924, −0.811, −0.591) also performed above Shore D hardness. On higher plasticizer contents (DOP40, DOP50), *S_d_* (−0.769, −0.884, −0.925) performed better than Shore D hardness. The best deducted quantities for monitoring Shore D hardness behavior are the following: CxLFxlgF (−0.816, −0.856, −0.832), CLF (−0.838, −0.876, −0.796), and Alog (−0.832, −0.873, −0.736). Surprisingly, the correlation to differences was low on DOP50 (best: *S_d_*, 0.413) and DOP40 (best: CF, 0.605) specimens. On DOP30 films, all quantities were below 0.7 correlation; only Alog (−0.698) was close to this level.

## 5. Discussion

### 5.1. PVC Cable Samples

The measurement results show that there are better deducted quantities for monitoring thermal aging than Shore D hardness. However, if we consider aging temperatures separately, the best parameters vary. Mechanical measurements (i.e., indenter-type, elongation at break, etc.) have proved to be useful for monitoring the degree of deterioration caused by thermal stress [9,22,28,29]. However, these studies focused on the long-term effect of aging, not short-term. Nevertheless, in [9], although the research did not focus on short-term aging and lower temperature (80 °C) was used in the first aging periods, an initially decreasing trend in Shore D hardness was measured. This trend converted to an increasing one afterward, and the same was inspected in our measurements. Therefore, for following the effect of short-term aging, Shore D hardness is not the best parameter to choose.

It can be stated that, on average, the best parameters tend to be Alog, CxFxLF, CxlgFxLF, CF, and CC. The first three of these are closely related to the dielectric loss in insulation caused by an AC stress that can be seen in Equation (7):

(7) $$P_{d}=E^{2}\varepsilon_{r}\varepsilon_{0}\omega \;tan\delta$$

where *P_d_* is the dissipated power, *E* is the electric field, *ε_r_ε_0_* is the relative permittivity times the vacuum permittivity, ω is the angular frequency, and *tanδ* is the loss factor. The close relationship lies in the fact that *tanδ* appears in Alog, CxFxLF, CxlgFxLF deducted quantities; the angular frequency is directly proportional to the frequency; and the capacitance is directly proportional to the permittivity. This means that as a result of short-term thermal aging, the dissipated energy generated by the 20 Hz–500 kHz frequency range increases. This result reinforces the result of a previous study in which the dissipated energy was found to correlate well to thermal aging using PVC samples in the case of long-term aging. The dissipation energy was calculated from the measured cumulative charge [8] so that the study used completely different parameters to measure.

Changes in central capacitance suggest that the capacitance of the cables increases by short-term thermal aging considering the 20 Hz–500 kHz frequency range. This effect is due to the change in molecular structure that results in relative permittivity change of the insulation. This effect was also inspected in [28] but with a wider frequency range, performing a significantly longer aging process (hundreds of hours) at a higher temperature (140 °C).

Another interesting result is that the central frequency almost linearly decreases by thermal aging. This means that the overall loss factor spectrum inspected in the 20 Hz–500 kHz range is shifting towards the lower frequencies by thermal aging. This was also inspected in [17,18], where the peak of tan δ values shifted towards the lower frequencies. However, the whole process could be seen by eye and not by one specific number. Moreover, the scope of those investigations was a lot smaller. Examining the results of [28], the correlation between aging time and central frequency seems to be dependent on the frequency range inspected. However, the range used in this study seems viable for the results published in [27] as well. Another study has shown that by aging PVC at 120 °C, the loss factor increases at 50 Hz frequency after 1500 h, and this time drops to 250 h at 140 °C [30]. Although the frequency dependence was not in the scope of that study, the increasing trend at a low frequency implies the central frequency shifting towards the lower frequency range.

The initial steepness of decay voltage, which is directly proportional to the specific conductivity of the insulating material, proved to be useful only at 140 °C. The reason for this could be that at this temperature, the plasticizer loss becomes the dominant process of aging in a shorter time than at lower temperatures. If the plasticizer content is higher, the conductivity, and therefore *S_d_*, will be also higher [25,26]. Hence, the results suggest that in short-term thermal aging, initially not the loss of plasticizer is the dominant aging process. Instead, the initial drop of Shore D hardness might be caused by the rearrangement of molecules and the aggregation of molecular chain also known as the annealing effect as claimed in [31,32]. Another possible explanation for the initially decreasing trend in hardness is that due to the elevated temperature, the plasticizer from the filling material migrates to the jacket, softening the material as proposed by [17,18,33].

The resting of samples for 2 months resulted in a breaking point in the measurement results. All measured data acted the same way after restarting the measurements as if a new measurement had been started. This implies that the measured data are dependent on the time passed between the aging rounds. One reason for this would be that the plasticizer loss is a two-step process, namely, the diffusion from the bulk of PVC to the surface and the evaporation from the surface to the atmosphere [34]. Since the aging was performed in a well-ventilated oven, the plasticizer loss was diffusion-controlled in our case. As a result of aging, as an earlier study has shown, the plasticizer content varies by the depth of the insulation [35] having less on the surface. Nevertheless, by resting the samples for a longer period, these differences can vanish. This explains the softening of the samples after the resting period. The reason for this is the migration of the plasticizing agent that is seeking the equilibrium of the plasticizer concentrate. Another reason could be the interdiffusion between the filling material or the core insulation and the jacket, as mentioned earlier.

### 5.2. PVC Films

The results of the PVC films have shown similar behavior to the PVC cables in terms of short-term thermal aging’s effect on hardness. The results have also shown that there are better deducted quantities for following short-term thermal aging than Shore D hardness. Nevertheless, due to the fact that in this case the aging temperature remained unchanged and the percentage of plasticizing agent varied, other conclusions can be derived from the results. The best deducted quantities were different from the ones in cables since these were CxFxLF, CC, CFxCLFxCC, and CFxCLF in the case of plasticized PVC specimens and Alog, CxFxLF, CxlgFxLF, CF, and CC in the case of PVC cables. At first sight, from the best results, only two (CxFxLF, CC) appear in both films and cables. However, if we take into consideration that a PVC cable insulation usually contains 40 or less wt% plasticizer content, CxlgFxLF and Alog also show a good correlation. By decreasing the plasticizer content, both CxlgFxLF and Alog reach higher than 0.9 correlations. In the case of cables aged at 110 °C, the best parameters were nearly the same: CFxCLF, CxFxLF, CFxCLFxCC, and CLF. In conclusion, only CLF and CC meant the difference between the best parameters in the case of cables and films.

The initial steepness of decay voltage correlated much better with aging time on specimens having higher plasticizer content (DOP0…DOP50): −0.535, −0.769, −0.884, and −0.925. This is again because of the fact that *S_d_* is directly proportional to the specific conductivity [21,25,26].

There is an initial drop in hardness even in nonplasticized PVC specimens, reinforcing the hypothesis that the root cause of this phenomenon is the annealing effect [31,32]. The annealing effect is the rearrangement of the molecules in the insulating material. Hence, the evaporation of the plasticizing agent is not the dominant process during the first aging period. On the other hand, in [32] the plasticizer concentration of the inspected samples dropped as a result of thermal aging even after 50 h of aging at 100 °C. Hence, it can be stated that at this level of aging, the dominant aging process is the annealing effect even though the plasticizer content of the inspected samples possibly also started to decrease because of thermal aging. The decreasing trend of *S_d_* values is also reinforcing this hypothesis. The initial drop of hardness might also be caused by the stearic acid lubricant, which has been used for sample preparation. This lubricant increases the glass transition temperature of PVC [36]. Since stearic acid is an external lubricant [37], after the first aging period, the lubricant might have evaporated from the surface region of the specimens. This could decrease the glass transition temperature and soften the PVC as well.

The month-long resting of the samples resulted in a behavior similar to the phenomenon already seen in the case of PVC cables: Shore D hardness did not increase immediately after the aging started. Instead, it initially decreased again and, after reaching a minimum value, converted to an increasing trend. The reason for this is that the plasticizer content varies by the depth of the insulation as a result of thermal aging. The samples contain less plasticizer on the surface [35] since the circumstances of aging (ventilated aging chamber) suggest a diffusion-controlled plasticizer loss.

It is important to note that the aging time needed to reach the initial hardness before the 1-month-long resting also differed by plasticizer content. This lasted for around 15, 9, and 6 h in the case of DOP50, DOP40, and DOP30 samples, respectively. This effect cannot be identified in nonplasticized samples. This supports the hypothesis that the plasticizer content of the PVC-insulating material is dependent on the time needed to reach the initial hardness in the case of short-term thermal aging.

## 6. Conclusions

The aim of this work was to find better quantities than Shore D hardness for monitoring short-term thermal aging on low-voltage PVC cables and PVC films. To achieve this goal, the effect of multiple aging temperatures (110, 125, 140 °C) on PVC cables and the effect of varying plasticizer contents (0, 30, 40, 50 wt% DOP) on PVC films were investigated.

This investigation has revealed for both cables and films that for following short-term thermal aging, Shore D is not the best parameter to choose. This is because hardness does not increase immediately by aging, but only after an initial drop because of the annealing effect in PVC. Instead, Alog, CxFxLF, CxlgFxLF, CF, and CC values are the best deducted quantities for following short-term thermal aging for cables. For plasticized PVC specimens, CxFxLF, CC, CFxCLFxCC, and CFxCLF performed best. However, in DOP40 samples (having about the same plasticizer content as cable sheaths), Alog and CxlgFxLF also appeared as the best choices. To sum up the results, Alog, CxFxLF, CxlgFxLF, and CC deducted quantities are the best choices for monitoring short-term thermal aging on PVC cable sheaths, providing four new quantities from nondestructive measurements.

Moreover, these results fit in and supplement the recent findings regarding thermal aging in PVC insulation, although approaching the problem from a different and new point of view. Utilizing the new deducted quantities, the aging process can be characterized by only one number from loss factor measurement, although a wider frequency range is measured.

The results presented in this paper provide a good basis for a better condition monitoring system that utilizes online measurements or in any case where the identification of a short-term thermal shock is helpful (e.g., in NPPs).

## Figures and Tables

**Table 1:** Aging times at 125 °C and 140 °C (row 1) and the calculated equivalent aging times at 110 °C (row 2) applying 80 kJ/mol (0.829 eV) activation energy.

	125 °C								140 °C			
Aging Time at Different Temperatures (h)	3	6	9	12	15	18	24	30	3	6	12	18
Equivalent Aging Time at 110 °C (h)	8	15	23	31	39	46	62	77	19	37	74	111

**Table 2:** Correlations between deducted quantities and equivalent aging time, hardness, and hardness changing of PVC cable samples.

		CLF	CF	CFxCLF	CFxCLFxCC	CxFxLF	CC	Alog	ShoreD	CxLFxlgF	*S*_d_
110 °C (21 meas.)	Aging t.	0.687	−0.853	−0.676	0.077	−0.354	0.874	0.875	0.853	0.849	−0.636
	ShoreD	0.332	−0.700	−0.717	−0.092	−0.592	0.706	0.627	1.000	0.598	−0.388
	Diff.	−0.831	−0.612	−0.897	−0.835	−0.844	−0.589	−0.753	1.000	−0.765	0.090
110 °C (9 meas.)	Aging t.	0.675	0.277	0.830	0.869	0.849	0.877	0.815	0.432	0.826	−0.440
	ShoreD	−0.319	0.842	0.407	0.357	0.016	0.293	−0.045	1.000	−0.013	0.065
	Diff.	−0.941	0.781	−0.025	−0.174	−0.896	−0.389	−0.869	1.000	−0.862	0.306
125 °C (9 meas.)	Aging t.	0.821	−0.898	−0.469	0.835	0.935	0.984	0.959	0.721	0.957	−0.393
	ShoreD	0.281	−0.519	−0.609	0.464	0.524	0.677	0.529	1.000	0.531	0.076
	Diff.	−0.739	0.586	−0.433	−0.336	−0.492	−0.167	−0.754	1.000	−0.707	0.302
140 °C (4 meas.)	Aging t.	0.700	−0.962	−0.980	−0.769	0.601	0.848	0.860	0.932	0.811	−0.980
	ShoreD	0.395	−0.811	−0.957	−0.943	0.279	0.606	0.619	1.000	0.548	−0.915
	Diff.	−0.993	0.793	−0.851	−0.990	−0.960	−0.940	−0.977	1.000	−0.975	−0.569

**Table 3:** Correlations between deducted quantities and equivalent aging time, hardness, and hardness changing of PVC films.

		CLF	CF	CFxCLF	CFxCLFxCC	CxFxLF	CC	Alog	ShoreD	CxLFxlgF	*S*_d_
DOP0	Aging t.	−0.580	−0.295	−0.516	−0.628	−0.865	−0. 15	−0.569	0.122	−0.686	−0.535
	ShoreD	0.404	−0.451	−0.377	−0.297	0.019	0.281	0.458	1.000	0.403	−0.776
	Diff.	0.947	−0.114	0.376	0.603	0.924	0.976	0.934	1.000	0.657	−0.904
DOP30	Aging t.	−0.950	−0.027	−0.811	−0.867	−0.935	−0.843	−0.924	0.810	−0.935	−0.769
	ShoreD	−0.838	0.418	−0.398	−0.468	−0.675	−0.660	−0.832	1.000	−0.816	−0.880
	Diff.	−0.693	0.746	0.023	−0.156	−0.549	−0.627	−0.698	1.000	−0.060	−0.808
DOP40	Aging t.	−0.879	−0.637	−0.831	−0.869	−0.929	−0.905	−0.811	0.724	−0.875	−0.884
	ShoreD	−0.876	−0.087	−0.362	−0.415	−0.620	−0.728	−0.873	1.000	−0.856	−0.423
	Diff.	−0.456	0.605	0.514	0.489	0.255	−0.066	−0.518	1.000	−0.414	−0.131
DOP50	Aging t.	−0.690	−0.829	−0.918	−0.936	−0.958	−0.950	−0.591	0.836	−0.771	−0.925
	ShoreD	−0.796	−0.544	−0.692	−0.721	−0.786	−0.830	−0.736	1.000	−0.832	−0.639
	Diff.	−0.364	0.055	−0.046	−0.038	−0.059	0.074	−0.250	1.000	−0.278	0.413
